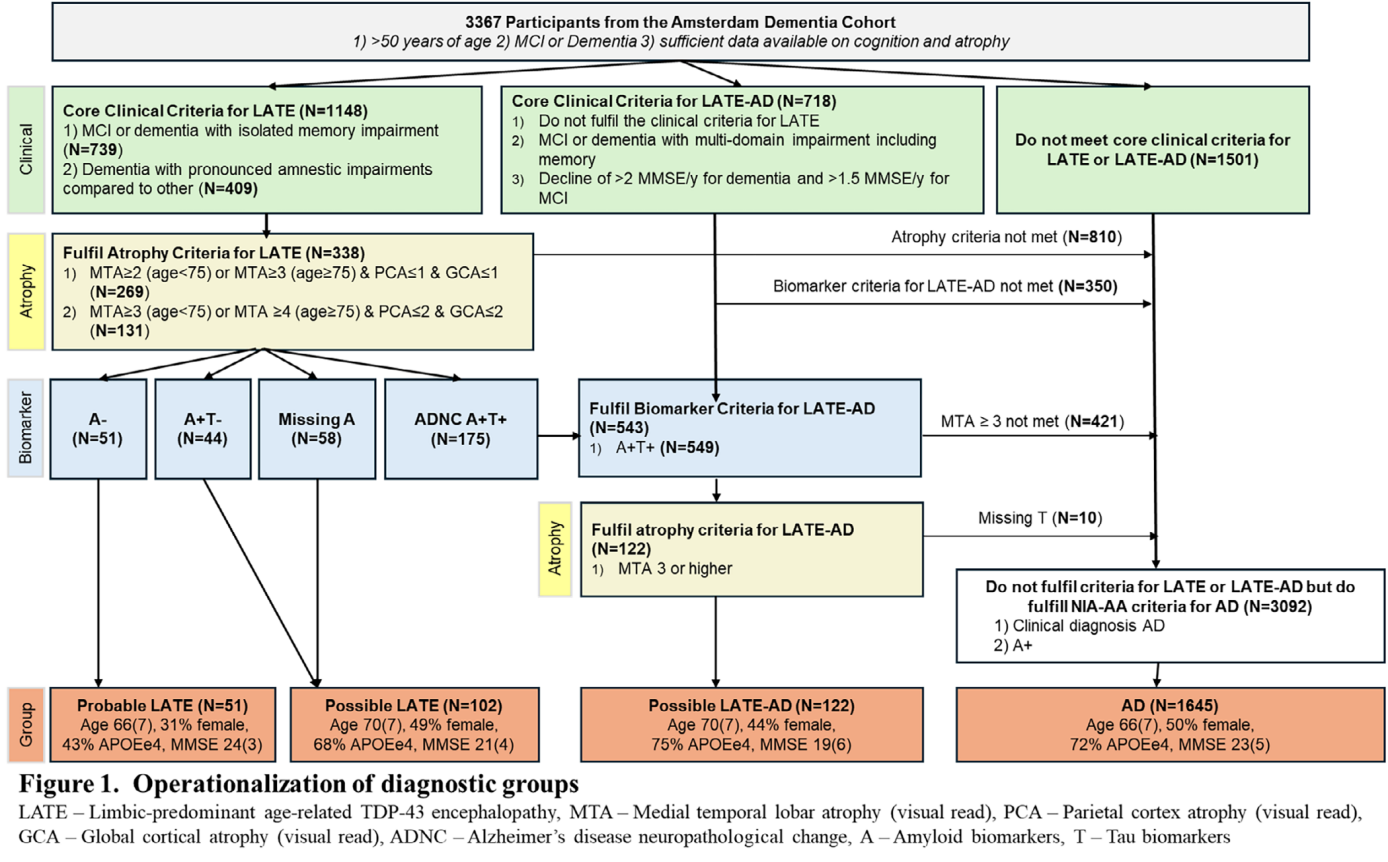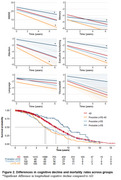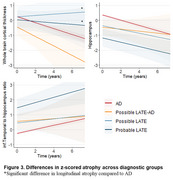# Characterization of individuals fulfilling clinical criteria for limbic‐predominant age‐related TDP43 encephalopathy (LATE) in a tertiary memory clinic

**DOI:** 10.1002/alz70856_104809

**Published:** 2026-01-07

**Authors:** Colin Groot, Ismael Luis Calandri, Ilse Bader, Diana I. Bocancea, Hannah de Bruin, Maria Carrigan, Suzie Kamps, Lotte A. de Koning, Sophie E. Mastenbroek, Roos M. Rikken, Bastiaan G J van Tol, Marie R. Vermeiren, Alex J. Wesseling, Ye Xia, Charlotte E. Teunissen, Elsmarieke van de Giessen, Frederik Barkhof, Laura E. Jonkman, Sven J van der Lee, Casper de Boer, Annemieke J.M. Rozemuller, Floor Duits, Betty M. Tijms, Wiesje M. van der Flier, Yolande A.L. Pijnenburg, Emma M. Coomans, Rik Ossenkoppele

**Affiliations:** ^1^ Alzheimer Center Amsterdam, Neurology, Vrije Universiteit Amsterdam, Amsterdam UMC location VUmc, Amsterdam, Netherlands; ^2^ Fleni, CABA, Buenos Aires, Netherlands; ^3^ Amsterdam Neuroscience, Brain Imaging, Amsterdam, Netherlands; ^4^ Amsterdam Neuroscience, Neurodegeneration, Amsterdam, Netherlands; ^5^ Alzheimer Center Amsterdam, Department of Neurology, Amsterdam Neuroscience, Vrije Universiteit Amsterdam, Amsterdam UMC, Amsterdam, Netherlands; ^6^ Institute for Stroke and Dementia Research, Klinikum der Ludwig‐Maximilians Universität München, Munich, Germany; ^7^ Alzheimer Center Amsterdam, Department of Neurology, Amsterdam Neuroscience, Vrije Universiteit Amsterdam, Amsterdam UMC, Amsterdam, North Holland, Netherlands; ^8^ Radiology & Nuclear Medicine, Vrije Universiteit Amsterdam, Amsterdam UMC location VUmc, Amsterdam, Netherlands; ^9^ Clinical Memory Research Unit, Department of Clinical Sciences Malmö, Faculty of Medicine, Lund University, Lund, Sweden; ^10^ Division of Clinical Geriatrics, Center for Alzheimer Research, Department of Neurobiology, Care Sciences and Society, Karolinska Institute, Stockholm, Stockholms län, Sweden; ^11^ Department of Radiology & Nuclear Medicine, Amsterdam UMC, Amsterdam, Netherlands; ^12^ Amsterdam UMC, location VUmc, Amsterdam, Netherlands; ^13^ Amsterdam UMC; locatie VUmc, Amsterdam, N‐H, Netherlands; ^14^ Neurochemistry laboratory, Clinical Chemistry, Amsterdam UMC location VUmc, Amsterdam, Netherlands; ^15^ Department of Radiology and Nuclear Medicine, Amsterdam UMC, Vrije Universiteit Amsterdam, Amsterdam Neuroscience, Amsterdam, Netherlands; ^16^ Amsterdam UMC, location VUmc, Amsterdam, Noord‐Holland, Netherlands; ^17^ Section Genomics of Neurodegenerative Diseases and Aging, Department of Human Genetics, Vrije Universiteit Amsterdam, Amsterdam, Noord‐Holland, Netherlands; ^18^ VU University Medical Center, Amsterdam, Netherlands; ^19^ Department of Pathology, Amsterdam Neuroscience, Amsterdam UMC, Amsterdam, Noord‐Holland, Netherlands; ^20^ Alzheimer Center Amsterdam, Department of Neurology, Vrije Universiteit Amsterdam, Amsterdam UMC location VUmc, Amsterdam, Netherlands; ^21^ Alzheimer Center, Department of Neurology, Amsterdam UMC, Vrije Universiteit Amsterdam, Amsterdam Neuroscience, Amsterdam, Netherlands; ^22^ Alzheimer Center Amsterdam, Department of Neurology, Amsterdam UMC, location VUmc, Amsterdam, Netherlands; ^23^ Department of Neurology, Alzheimer Center Amsterdam, Amsterdam Neuroscience, Vrije Universiteit Amsterdam, Amsterdam, Netherlands; ^24^ Amsterdam University Medical Center, Amsterdam, Netherlands

## Abstract

**Background:**

Limbic‐predominant age‐related TDP‐43 encephalopathy (LATE) clinically mimics and often co‐occurs with Alzheimer's disease (AD). Expert consensus criteria have been proposed for the LATE clinical diagnosis, integrating clinical and radiological features, and AD biomarkers. Here, we applied the newly proposed criteria in a tertiary memory clinic population.

**Method:**

We included participants from the Amsterdam Dementia Cohort aged >50 years who received a diagnosis of MCI or dementia between 1997‐2024. Following the LATE consensus criteria scheme (Figure 1), we categorized participants as “Probable LATE”, “Possible LATE” or “Possible LATE‐AD” (i.e. LATE clinical and radiological profile with AD biomarker profile). Participants not fulfilling criteria for LATE but fulfilling NIA‐AA criteria for AD were categorized as AD. We compared the LATE groups with AD on cognitive decline (*N* = 1046, N Mean time=2.7[1.8] years) and atrophy (*N* = 208, Mean time=2.1[1.6]) using linear‐mixed effects models, and on mortality rates using Cox proportional hazard models.

**Result:**

Of the 3367 individuals, 1920 were classified into one of the four groups. Fifty‐one (1.5%) were classified as Probable LATE, 102 (3.0%) as Possible LATE, 122 (3.6%) as Possible LATE‐AD, and 1645 (48.8%) as AD (Table 1). Compared to AD, Probable LATE showed an attenuated cognitive decline (b[SE] for MMSE=0.12[0.05], *p* = 0.02) and lower mortality rates (HR[95% CI]=0.75[0.58‐0.95], *p* = 0.02), while individuals with Possible LATE‐AD had faster cognitive decline (b for MMSE=‐0.12[0.05], *p* = 0.01) and higher mortality rates (HR=1.55[1.25‐1.92], *p* <0.001, Figure 2). Compared to AD, Probable LATE had, at baseline, lower hippocampal volumes (b=‐0.83[0.27], *p* <0.01), and higher inferior‐temporal to hippocampal volume ratios (b=0.81[0.27], *p* <0.01). Furthermore, in Probable LATE, atrophy in a whole‐brain region‐of‐interest was slower compared to AD (b=0.14[0.08], *p* = 0.04). Possible LATE‐AD had, at baseline, thinner whole‐brain cortex (b=‐0.69[0.30], *p* = 0.02), lower hippocampal volumes (b=‐1.54[0.31], *p* <0.01), and higher inferior‐temporal to hippocampal volume ratios (b=1.73[0.30], *p* <0.01) than AD, but there was no difference in atrophy rates between Possible LATE‐AD and the other groups (Figure 3).

**Conclusion:**

In a tertiary memory clinic population, the newly proposed clinical LATE criteria reveal clinical and atrophy trajectories that are distinct from AD, especially for Probable LATE and Possible LATE‐AD. Differential clinical and biological disease trajectories highlight the relevance of the LATE classification for diagnostic and prognostic purposes